# Research into Morphofunctional Characteristics of Erythrocytes in COVID-19 Patients

**DOI:** 10.3390/biomedicines10030553

**Published:** 2022-02-25

**Authors:** Victor Revin, Larisa Balykova, Sergey Pinyaev, Ilya Syusin, Olga Radaeva, Nadezhda Revina, Yuliya Kostina, Evgeniy Kozlov, Vera Inchina, Igor Nikitin, Alexandr Salikov, Ilya Fedorov

**Affiliations:** 1 Federal State Budgetary Educational Institution of Higher Education, Ogarev Mordovia State University, 430005 Saransk, Russia; revinvv2010@yandex.ru (V.R.); larisabalykova@yandex.ru (L.B.); ilya.sysin@gmail.com (I.S.); radaevamed@mail.ru (O.R.); nadyarev@yandex.ru (N.R.); bazunova.2013@mail.ru (Y.K.); dr.kozlov@yandex.ru (E.K.); v.inchina@ya.ru (V.I.); 2 Federal State Autonomous Institution, Treatment and Rehabilitation Center of the Ministry of Health of the Russian Federation, 125367 Moscow, Russia; igor.nikitin.64@mail.ru; 3 State Budgetary Institution of Healthcare, City Clinical Hospital after V.M. Buyanov of the Moscow Department of Health, 115516 Moscow, Russia; gkb12@zdrav.mos.ru (A.S.); rsmu@rsmu.ru (I.F.)

**Keywords:** erythrocytes, novel coronavirus infection, COVID-19, hemoglobin, Raman spectroscopy, laser interference microscopy

## Abstract

In this paper, the erythrocytes of healthy donors and people with a confirmed diagnosis of COVID-19 were tested by Raman spectroscopy and laser interference microscopy. We argue that erythrocytes (red blood cells) in COVID-19 patients have an irregular shape, and their morphometric characteristics are impaired in comparison to healthy red blood cells. Raman spectroscopy also allows us to detect a decreased oxygen transport function of these erythrocytes. The combination of these methods—Raman spectroscopy and laser interference microscopy—is a highly effective method for the diagnosis of SARS-CoV-2.

## 1. Introduction

The situation with the novel coronavirus infection, COVID-19, despite the unprecedented measures taken by the international medical community, remains distressing. Russian experts have developed and put into practice 12 versions of methodological recommendations for the diagnosis and treatment of novel coronavirus infection [1]. Each of them, based on an in-depth analysis of current scientific findings and the current epidemiological situation, administers the provision of medical care to patients with SARS CoV-2 infection with the maximum level of evidence.

Hematological disorders, such as thrombocytopenia and lymphopenia with a relatively high neutrophil count and an increased ratio of neutrophils to lymphocytes, are common signs of a severe course of a novel coronavirus infection, while their effects on the state of red blood cells and hemoglobin properties have not yet been studied [2].

It is probable that antibodies produced by plasma cells destroy erythrocytes, and nonstructural proteins (ORF1a, ORF10, ORF3a, and ORF8) disrupt hemoglobin’s oxygen transport function, displacing ferrous iron atoms from the heme’s porphyrin macrocycle [3].

Systemic inflammation with increased synthesis of IL-6, as well as interferon-γ, IL-1β, IL-33 and TNF, is the basis of the progressive course of COVID-19 and seriously affects erythropoiesis through various mechanisms supported in part by abnormal iron metabolism [4]. Pro-inflammatory cytokines inhibit erythroid progenitor cells, changing their morphology and reducing the lifespan of erythrocytes.

Thus, analysis of blood smears obtained from patients with anemia associated with COVID-19 showed several abnormalities in the shape of the red blood cells. The most common finding was the presence of a high frequency of stomatocytes and knizocytes, which are not often found on blood smears in other types of anemia. In line with recent studies, it can be hypothesized that RBC damage occurs as a result of immune-mediated mechanisms and/or physical damage to cells due to COVID-19 microangiopathy. The loss of RBC biconvexity and complement activation observed in COVID-19 may contribute to RBC accumulation and spontaneous agglutination and possibly contribute to microvascular thrombosis typical of COVID-19 [5].

Abnormal iron metabolism may be one of the factors impairing the oxygen transport properties of hemoglobin, so in the work of Ana C. Moreira et al., the authors combine low serum iron, low transferrin and high IL-6 levels as important predictors of disease severity in COVID-19. The change in serum ferritin levels over time, they report, should be considered as an indicator of disease progression [6].

Taking into account the possible role of the erythrocyte-dependent link in the pathogenesis of COVID-19, as well as the fact that erythrocytes and the entire circulatory system are constituents of the human body that respond quickly to external influences, including viral infection, it was of interest to make a comparative analysis of the state of oxygen transport function and morphometric indicators of erythrocytes in healthy and COVID-19-infected people. This was our main goal, and following up on this goal, we set out to complete a number of tasks:

(1) To study the oxygen transport properties of hemoglobin in patients with COVID-19;
(2) To make a comparative analysis of morphometric parameters of erythrocytes in healthy people and COVID-19 patients;
(3) To reveal the characteristics of erythrocytes and hemoglobin that change with the development of the disease and to determine whether they can serve as a diagnostic test in the case of the stability of indicators for all those who have had this disease.

## 2. Materials and Methods

### 2.1. Patients

This study enrolled 15 patients (7 males and 8 females, age from 40 to 86 years, 95% confidence interval (CI) (57–82%)) who were hospitalized with a novel coronavirus infection at Katkov Republican Clinical Hospital (Saransk, Republic of Mordovia) and underwent inpatient treatment from March 2021 to May 2021. Eligibility criteria were age between 18 and 80 years old, confirmed diagnosis of “moderate–severe course of coronavirus infection” and written informed consent. The diagnosis was established after receiving clinical, epidemiological and radiological findings (typical lesion of the lung parenchyma revealed by means of computed tomography) and positive PCR test results.

Ineligibility criteria were COVID-19 of mild course at the time of hospitalization, admission to the hospital after the 7th day of the disease and severe concomitant pathology (type 1 or type 2 diabetes mellitus of severe course at the stage of decompensation, stage III hypertension, bronchial asthma, liver and kidney failure at the stage of decompensation, grade III–IV chronic heart failure).

A suspension of erythrocytes was obtained from the whole venous blood of patients with COVID-19 on the first day of hospitalization before the appointment of combined therapy, following the Temporary Guidelines (version 12). Sodium citrate was used as an anticoagulant at a final concentration of 130 mm and a pH of 7.4. Blood was centrifuged for 15 min at 1500× *g* in a centrifuge. After that, the infusion fluid containing plasma and leukocytes was removed, and the erythrocytes were washed three times with a cooled phosphate buffer pH = 7.4 (2.0 g KCl; 2 g KH_2_PO_4_×H_2_O; 80 g NaCl; 15.6 g Na_2_HPO_4_×12H_2_O per 1 L of distilled water; pH was adjusted to 10 N NaOH).

All studied patients were divided into 3 groups by age:

Group 1: average age 53.6 years (*n* = 5), age range 42–61.
Group 2: average age 71.4 years (*n* = 5), age range 65–76.
Group 3: average age 85.7 years (*n* = 5), age range 81–86.

The study was approved by the Ethics Committee of the Ogarev Mordovia State University (Protocol No. 85 dated 27 May 2020). All patients signed voluntary informed consent. Biological material was obtained for research (blood) was carried out taking into account the provisions of the Helsinki Declaration of the WMA (2013) and the Protocol of the Council of Europe Convention on Human Rights and Biomedicine (1999), as well as the additional protocol of the Convention on Human Rights and Biomedicine in the field of biomedical research (2005).

### 2.2. Control Group

There was also a control group consisting of 10 practically healthy donors (aged 40–60 years), who had periodic health examinations. These donors were selected in such a way that their gender and age corresponded to the parameters of patients with COVID-19 in the other studied groups. Their average hematological parameters: RBC was 4.6 ± 0.06 × 10^12^/L; Hb was 136.1 ± 4.24 g/L.

### 2.3. Laser Interference Microscopy

Morphological changes and redistribution of hemoglobin in the cytoplasm were examined by laser interference microscopy using a laser interference microscope (LIM, All-Russian Research Institute of Optical and Physical Measurements, Moscow, Russia) based on the Linnik MII-4 apparatus. We used a microinterferometer (LOMO, Russia) with a lens of 30 × (NA = 0.65); the laser power on the object was less than 2 mW (λ = 650 nm). To obtain images, a VS-415U CCD video camera (NPK Videoscan, Russia) with a matrix size of 6.5 × 4.83 mm and a resolution of 782 × 582 pixels was used. The software WinPhast (All-Russian Research Institute of Optical and Physical Measurements, Moscow, Russia) was used to restore the phase image. To analyze the morphometric parameters of erythrocytes, the average phase height (the average value of the optical path difference (OPD)) surface area, cell thickness and phase volume were logged using FIJI software (ImageJ, USA). At least 100 cells from each sample were used for statistical processing of the results.

### 2.4. Raman Spectroscopy

The change in the oxygen transport properties of hemoglobin was studied by means of Raman spectroscopy (RS) on a Renishaw inVia Basis spectrometer with a short-focus extreme aperture lens monochromator (focal length no more than 250 mm). A laser was utilized to excite the spectra (radiation wavelength 532 nm, maximum radiation power 100 mW, lens 100×). The data logger was a CCD detector (1024 × 256 pixels with Peltier cooling up to −70 °C) with a grid of 1800 sl/mm. The digitized spectra were processed in WIRE 3.3 software. Baseline correction and smoothing of spectra were performed.

The change in the oxygen-binding properties of erythrocytes was tracked by the following Raman spectra characteristic bands (maximum positions indicated): 1355, 1375, 1550, 1580, 2850, 2880 and 2930 cm^−1^. It is known that their correlations allow for the prediction of various conformations of hematoporphyrin (HP) and, accordingly, the ability to bind and transport oxygen, analyzing, regardless of the hemoglobin content in a particular sample, the conformation of HP in deoxyhemoglobin (d–Hb) and its ability to bind ligands, as well as the conformation of HP in oxyhemoglobin (o–Hb) and its ability to dump oxygen. In this work, we relied on the following options to analyze RS data:

- The ratio of the Raman intensity bands 1375 cm^−1^–1355 cm^−1^ (I_1375_/I_1355_) of the blood, characterizing the relative number of hemoglobin complexes with ligands, mainly the amount of oxyhemoglobin (GB-O_2_);
- The ratio of the Raman intensity bands I_1355_/I_1550_ of the blood and the relative ability of hemoglobin in the sample to bind ligands (including oxygen);
- The ratio of the Raman intensity bands (I_1355_/I_1550_)/(I_1375_/I_1580_) of the blood and the affinity of hemoglobin to ligands, primarily to oxygen;
- The ratio of the Raman intensity bands I_1375_/(I_1355_ + I_1375_) of the blood, which indicates the relative amount of o-Hb in the blood;
- Raman bands I_1580_/I_1550_ of the blood and the contribution of vibrations of methine bridges in hematoporphyrin (characterizes the affinity of Hb to ligands, particularly to oxygen);
- Raman bands I_2850_/I_2880_ of the blood and the contribution of symmetric fluctuations to asymmetric fluctuations of amino acid methylene groups;
- Raman bands I_2930_/I_2850_ of the blood and the contribution of vibrations of symmetrical terminal methylene groups to symmetrical vibrations of amino acid methylene groups (characterizes the change in the polarity of the amino acids surrounding).

### 2.5. Statistical Analysis

The statistical significance of the mean values was calculated using Student’s *t*-test after checking the normality of the distribution of the studied parameters with Statistica 6.0. software. The value of correlation was estimated using the Pearson correlation coefficient. The significance of differences was assessed by Student’s *t*-test. The differences were considered significant at *p* ≤ 0.05. The research results are presented as the mean and standard deviation (mean ± SD).

Statistical data processing was performed using certified statistical software (StatSoft Statistica 10.0, IBM SPSS Statistics 22, Armonk, NY, USA). The quantitative data are presented drawing on the number of observations, arithmetic mean, 95% confidence interval (CI) for the mean, standard (mean square) deviation, median and the 5th and 95th percentiles. Ordinal, categorical and qualitative data are presented in the form of absolute frequencies (number of observations), relative frequencies (percentages) and 95% CI. The normality of the distribution was tested against Shapiro–Wilk or Kolmogorov–Smirnov criteria. In the case of a non-Gaussian distribution, nonparametric estimation methods (Wilcoxon–Mann–Whitney test) were used to compare the indicators.

## 3. Results and Discussion

During the hospital trial, the medical characteristics of 15 patients with a diagnosis of COVID-19 with bilateral multisegmental pneumonia, moderate course, were obtained at the time of hospitalization.

**Figure 1:**
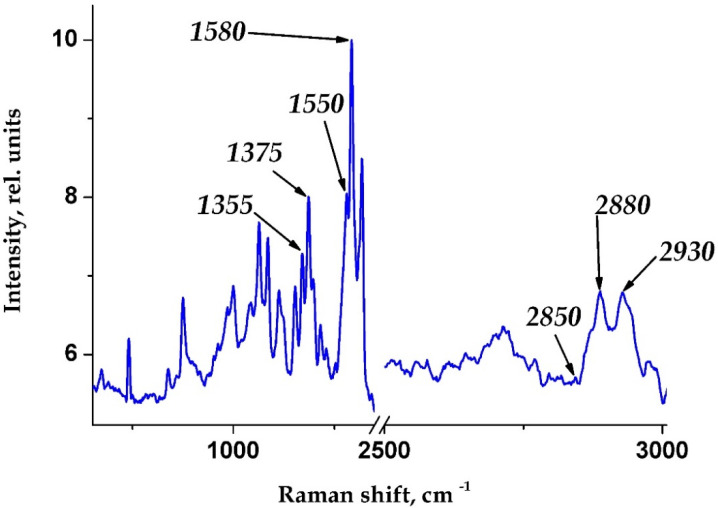
Raman spectrum of hemoglobin hematoporphyrin obtained from erythrocytes of a healthy person (control group).

Figure 1 shows a typical Raman spectrum of hematoporphyrin of human hemoglobin.

The most significant cause of disturbance in the oxygen transport system may be a change in the conformation of hemoglobin hematoporphyrin, as well as its ability to bind oxygen in hemoglobin. The position and intensity of the Raman scattering (RS) bands of the hemoglobin spectrum depend on variations in the bonds in the porphyrin ring; this makes it possible to evaluate hematoporphyrin (HP) conformation, which is directly related to the oxygen-binding properties of hemoglobin [7].

To analyze the conformation of hematoporphyrin hemoglobin (HP), we took into account the specific bands of the RS spectrum. In this article, to analyze the conformation and properties of the O_2_ binding of Hb, we used the following spectra of blood RS bands (the maximum positions are indicated): 1355, 1375, 1550 and 1580 cm^−1^ to characterize heme, the non-protein part of hemoglobin, and 2850, 2880 and 2930 cm^−1^ to characterize globin, the protein part of hemoglobin (Figure 1) [8].

**Figure 2:**
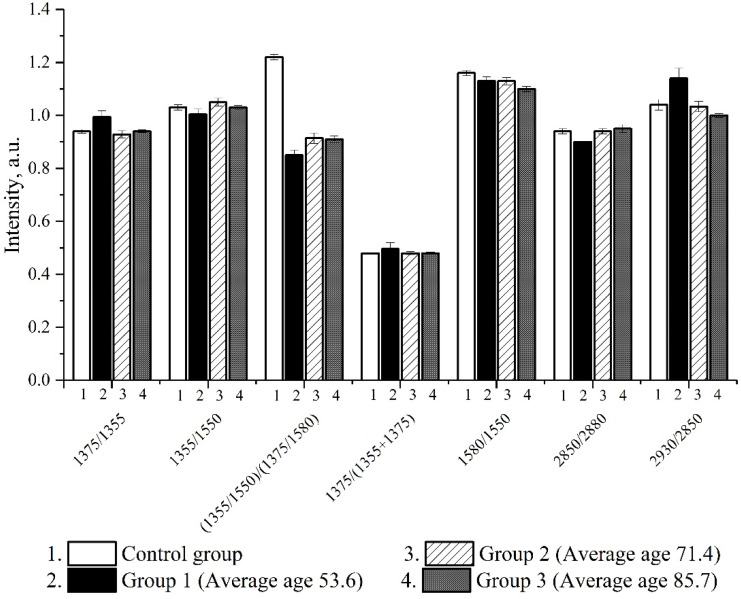
Changes in hematoporphyrin conformation and the ability of hemoglobin to transport oxygen in COVID-19 patients of different age groups.

During the analysis of the parameters of erythrocyte morphology and hemoglobin conformation in patients with COVID-19 of all age groups at the time of hospitalization, the following indicators decreased: (I_1375_/I_1355_), *p* < 0.01, characterizing the relative number of oxyhemoglobin complexes, and (I_1355_/I_1550_)/(I_1375_/I_1580_), *p* < 0.01, reflecting the affinity of hemoglobin to oxygen against a control group of individuals (Figure 2).

In the next series of experiments, using the method of laser interference microscopy, we studied morphometric parameters in patients with a novel coronavirus infection. There was a decrease in all analyzed indicators characterizing the morphofunctional characteristics of erythrocytes (erythrocyte area, optical path difference, thickness, phase volume and Hb packing density).

**Figure 3:**
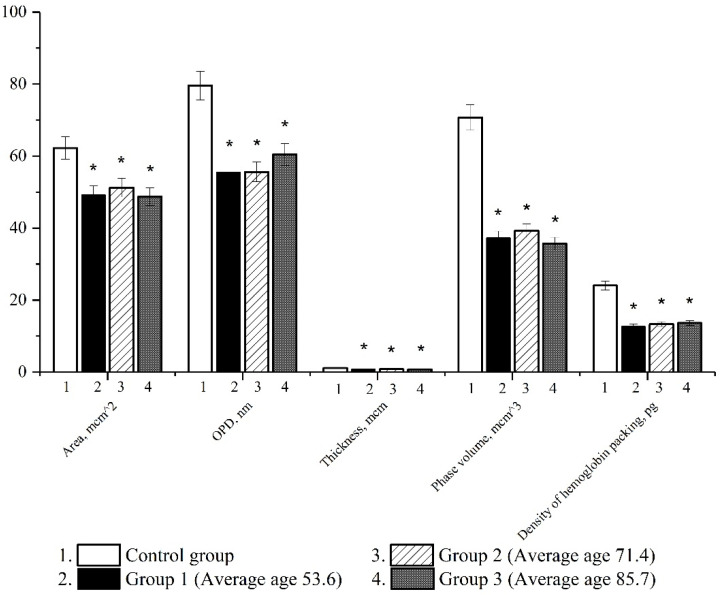
Study of the erythrocyte morphology of the control group and different age groups during the development of COVID-19 (* *p* ≤ 0.05 is the change in relation to the control).

The results point to profound changes occurring in red blood cells in patients with COVID-19. In patients of all age groups, the area, the distribution of hemoglobin, the optical path difference and the density of hemoglobin packing decreased (Figure 3).

**Figure 4:**
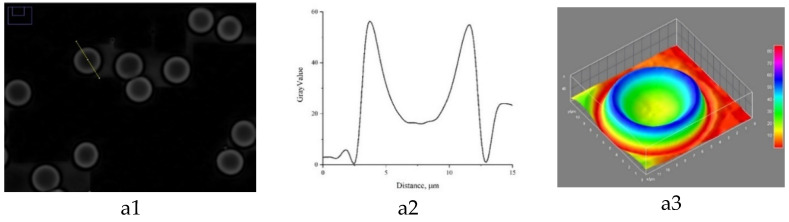

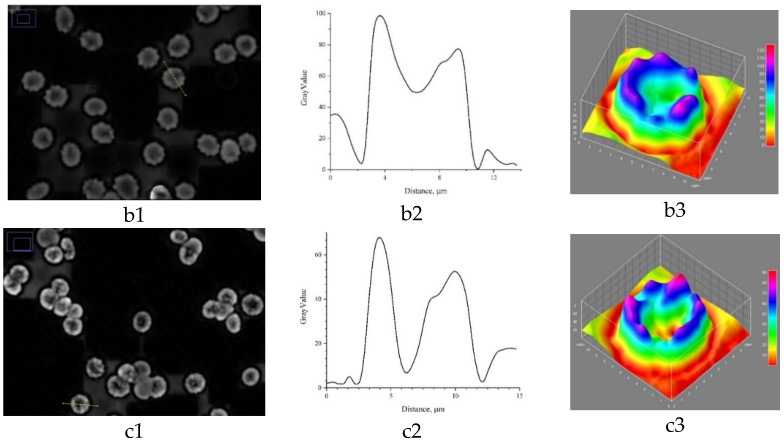
Image of a red blood cell from a healthy donor—(**a**). Image of a red blood cell from a person with a confirmed diagnosis of COVID-19—(**b**,**c**). (**1**)—erythrocyte phase image; (**2**)—erythrocyte profile; (**3**)—3D model of erythrocyte phase image.

LIM allowed us to obtain 3D models of the erythrocyte phase image of the control group, as well as the other groups. The data are shown in Figure 4.

With the development of the disease, there is a redistribution of intracellular contents, namely, hemoglobin, and the data in Figure 3 also prove this (Hb packing density), which in turn leads to the disorder of erythrocytes’ oxygen transport functions. The 3D model of the phase image of erythrocytes in patients diagnosed with a novel coronavirus infection has irregularities and surface roughness. Based on the data we obtained, the uniform distribution of hemoglobin and cytoplasm in erythrocytes was observed to be disturbed in pathology (Figure 3). The formation of local cytoplasmic clusters with an increased hemoglobin content was found. The revealed changes in the distribution of hemoglobin may cause the development of hypoxia due to the uneven redistribution of oxygen and carbon dioxide molecules during the hemodynamics of cells in blood vessels.

We found that patients with an extremely severe course of COVID-19 on the fifth day of inpatient treatment, whose condition was earlier (at the time of hospitalization) assessed as moderate, predictably had low values of the following parameters: the area and thickness of erythrocytes, the distribution of Hb and the density of hemoglobin packaging (*p* < 0.001), as well as the hemoglobin conformation index (I_1355_/I_1550_)/(I_1375_/I_1580_), reflecting its affinity to ligands (primarily to oxygen). This may point to the prognostic value of these morphofunctional characteristics of erythrocytes and hemoglobin in patients with COVID-19.

## 4. Discussion

The pandemic caused by SARS CoV-2 has caused enormous damage to all aspects of society. Unfortunately, the number of cases and the death toll are not decreasing; therefore, determining disease progression factors is important for risk stratification and the optimization of patient management tactics. Recent studies argue that a possible additional factor in the pathogenesis of the disease may be attributed to erythrocyto- and hemoglobino-pathies, aggravating hypoxemia and hypoxia, as well as an overload of cells with iron [9].

The changes in the morphology of erythrocytes that we found are likely accompanied by changes in the activity of membrane-bound enzymes and the functioning of membrane glycoproteins, which causes a redistribution of charges on the surface of erythrocytes and can lead to increased aggregation of erythrocytes and further deterioration of the rheological properties of blood and vascular endothelium function, including pulmonary pneumonitis [10].

Apart from morphological changes, patients with severe COVID-19 have a decreased number of erythrocytes. A meta-analysis of seven studies involving 9912 patients showed a significant connection of anemia with severe forms of COVID-19 (OR 2.44 (95% CI 1.75–3.40), *p* < 0.00001) [11].

A two-component mechanism of anemia may develop. Firstly, SARS-CoV-2 can interact with hemoglobin molecules on the erythrocyte via ACE2, CD147 and CD26 receptors and be conducive to viral damage of heme on the 1-β hemoglobin chain with possible hemolysis of erythrocytes. Secondly, SARS-CoV-2 can mimic the action of hepcidin, causing a deficiency of serum iron and hemoglobin. The resulting hyperferritinemia facilitates the activation of oxidative stress and lipid peroxidation, which can enhance the inflammatory response (and, together with activated macrophages, provoke a cytokine storm) and, thus, increase the severity of the disease [3].

The above theory has been criticized due to the lack of direct evidence of changes in hemoglobin properties under the influence of SARS CoV-2. In our work, a significant decrease in the affinity of hemoglobin to oxygen was revealed in patients with a severe form of the disease, which was clinically accompanied by a high oxygen demand with “stringent parameters” of ALV (artificial lung ventilation) in two out of four patients with an extremely severe form of the disease.

Other studies of the erythrocytes of 23 healthy donors and 29 patients diagnosed with COVID-19 revealed elevated levels of glycolytic products and impaired lipid metabolism in the erythrocytes of the COVID-19 patients. The authors believe that the functional consequences of such disorders can be unpredictable [12].

A number of authors argue that damage to erythrocytes in cases of COVID-19 occurs due to immune-mediated mechanisms and/or damage to cells induced by microangiopathy. The loss of erythrocytes’ bi-concavity and complement activation observed in COVID-19 may contribute to spontaneous agglutination of erythrocytes and possibly microvascular thrombosis typical of COVID-19. It is possible that sepsis, hypoxia and immune disorders (antibodies binding erythrocytes) affect the morphology, rheology and survival of erythrocytes, adding to the complex pathogenesis of COVID-19 [5].

Several groups of authors proposed to introduce to clinical practice indicators such as red cell distribution width (RDW) increase during the risk stratification of patients with COVID-19. Approximately 19.6% of patients with COVID-19 in the study by Rapp JL and co-authors (2021) had increased RDW at the time of diagnosis, which was statistically significantly related with mechanical ventilation (*p* = 0.0109) and mortality (*p* < 0.0001) [13,14].

We established a relationship between the thickness of erythrocytes and the density of hemoglobin packing in erythrocytes with the clinical characteristics of the disease (in particular, with the volume of involvement of the lung parenchyma). Clearly, these values of erythrocyte morphology are more helpful in predicting the severe course of the disease, owing to the simplicity and reliability of estimation and the reproducibility of the technique.

The changes in the conformation of hematoporphyrin and hemoglobin oxygen-binding properties are indicative of the disorder in the functional activity of erythrocytes, which works toward the aggravation of hypoxia.

Our research also argues that the low content of oxyhemoglobin complexes also correlates with the severity of COVID-19, particularly with the level of saturation on the fifth day of treatment, and the defining of hemoglobin conformation by means of Raman spectroscopy can be used to determine the likelihood of disease progression.

Recapitulating our findings, we can say that one of the important reasons for the impaired supply of oxygen to the human body is the change not only in the structure of the lung tissue but also in the functional characteristics of hemoglobin, which dramatically reduces the efficiency of oxygen binding and leads to a sharp shortage of oxygen in organs and tissues. It can also be confidently stated that the parallel use of two methods, Raman spectroscopy and laser interference microscopy, will not only allow us to predict the course of the disease but will also be an effective and perhaps the fastest diagnostic test for the detection of COVID-19.

## Data Availability

The data used to support the findings of this study are included within the article.
